# From Plastic Waste to Power Source: Polyethylene-Derived Anodes for Lithium-Ion Batteries

**DOI:** 10.3390/nano16201310

**Published:** 2026-10-16

**Authors:** Kuanysh Kurtibay, Yryszhan Tashenova, Aisha Zhanaikhan, Kuralay Korzhynbayeva, Aliya Mukanova, Arailym Nurpeissova

**Affiliations:** Institute of Batteries (IoB), Kabanbay Batyr Ave. 53, Astana 010000, Kazakhstan; kurtibayqb@gmail.com (K.K.); iriszhan9696@gmail.com (Y.T.); aisha.zhanaikhan@gmail.com (A.Z.); kuralay987@gmail.com (K.K.); aliya.mukanova@nu.edu.kz (A.M.)

**Keywords:** plastic waste upcycling, polyethylene, sulfonation-assisted carbonization, disordered carbon, anode material, lithium-ion battery

## Abstract

Polyethylene, the largest component of plastic waste, decomposes on heating almost entirely to volatiles rather than to carbon. Here, one post-consumer low-density polyethylene (LDPE) film and one high-density polyethylene (HDPE) bottle were converted into disordered carbons by sulfonation-assisted carbonization. Sulfonation raised the carbon residue at 900 °C from 1.21 to 32.63 wt.% for LDPE and from 0.06 to 8.82 wt.% for HDPE. The two carbons are indistinguishable by X-ray diffraction and high-resolution transmission electron microscopy (interlayer spacing 0.365–0.366 nm, crystallite thickness ≈1.4 nm), have the same Raman disorder ratio (1.01–1.02) and retain little sulfur (≤0.51 at.%), but differ in specific surface area (53.7 and 32.5 m^2^ g^−1^). As lithium-ion anodes, the LDPE- and HDPE-derived carbons deliver 338 and 302 mA h g^−1^ after 100 cycles at 100 mA g^−1^, with capacity still rising, and 155 and 130 mA h g^−1^ at 2 A g^−1^. The larger first-cycle loss and higher capacity of the LDPE-derived carbon correlate with its larger surface area, consistent with the precursor acting through its response to the sulfonation pretreatment rather than through carbonization. These results are a proof of concept; extension to other and mixed waste streams requires further study.

## 1. Introduction

The escalating accumulation of plastic waste in the environment represents one of the most pressing global sustainability challenges of the 21st century [1]. Among commodity polymers, polyethylene (PE)—in its low-density (LDPE) and high-density (HDPE) forms—constitutes a major fraction of municipal plastic waste, owing to its extensive use in packaging, consumer goods, and industrial applications [2]. Conventional disposal routes, including landfilling and incineration, are increasingly untenable from both environmental and regulatory perspectives, while mechanical recycling is constrained by degradation-induced property deterioration and the practical difficulties of sorting mixed polyolefin streams [3]. These limitations have motivated intensive research into chemical upcycling strategies that can convert plastic waste into value-added materials, thereby closing the carbon loop and contributing to a circular plastics economy [4,5].

At the same time, lithium-ion batteries (LIBs) remain the dominant technology for portable electronics, electric vehicles and stationary storage, and their further development depends not only on the intrinsic capacity of the electrode materials but also on how ions and electrons are transported through the electrode and across its interfaces [6,7,8]. Poorly formed electrode–electrolyte interfaces hinder ion transport and promote side reactions that shorten cycle life [6], whereas incomplete electrolyte wetting and long ionic pathways limit the fraction of active material that is utilized, particularly in thick, high-loading electrodes [7,8]. For carbon anodes, the accessible surface area, porosity and surface chemistry of the active material therefore matter alongside its bulk structure. Low-cost carbons that meet these requirements are increasingly sought from waste feedstocks, including biomass residues such as olive pomace [9] and plastic waste such as polypropylene packaging and acrylonitrile–butadiene–styrene (ABS) printing scraps [10,11].

Within this context, the thermochemical conversion of waste plastics into carbon materials has emerged as a particularly attractive pathway [12]. The inherently high carbon content of polyolefins, combined with the absence of heteroatoms that complicate purification, renders PE an appealing precursor for the synthesis of functional carbons [13]. Numerous studies have demonstrated that carbonization of PE under inert atmospheres yields disordered carbonaceous solids with tunable microstructures; these materials have found applications as catalyst supports, adsorbents, and, notably, as anode materials for LIBs [14]. The electrochemical performance of such carbons in LIBs is governed by a complex interplay of structural parameters—including interlayer spacing, crystallite dimensions, and the density of defect sites—all of which are sensitive to the processing conditions, particularly the carbonization temperature, heating rate, and duration, of thermal treatment [10,15,16].

A critical and largely unresolved question concerns the extent to which the properties of the resultant carbon depend on the specific characteristics of the PE precursor. LDPE and HDPE, though chemically identical in terms of repeating ethylene units, differ substantially in their macromolecular architecture: LDPE possesses a highly branched structure with both short- and long-chain branches, whereas HDPE is essentially linear with minimal branching [17]. These architectural differences manifest in distinct physical properties, including density, crystallinity, melting behavior, and chain mobility during thermal degradation: LDPE typically exhibits 40–60% crystallinity and a density of 0.917–0.930 g cm^−3^, while HDPE reaches 70–90% crystallinity and a density of 0.94–0.96 g cm^−3^ [18]. It remains unclear, however, whether such precursor-specific features survive the carbonization process to influence the structural and functional properties of the final carbon product, or whether the high-temperature treatment obliterates any memory of the original polymer architecture. This question also has practical implications: if the carbonization outcome were insensitive to the PE grade, the need to separate polyolefin streams before conversion could be reduced [19,20], although such a possibility must ultimately be tested on multiple and mixed waste streams.

Prior investigations into the carbonization of polyolefins have predominantly employed either model polymers or virgin resins, with relatively few studies addressing real-world waste streams that inherently contain additives, contaminants, and degradation products. Furthermore, the majority of reported syntheses rely on direct pyrolysis without pre-carbonization chemical treatment, often yielding carbons with limited structural order and modest electrochemical performance [21]. More recent efforts have explored sulfonation to introduce crosslinking and stabilize the polymer backbone prior to carbonization, thereby enhancing carbon yield and promoting the development of turbostratic domains [22]. Treatment with sulfuric acid cross-links the chains into an infusible network, so that a polymer which otherwise volatilizes almost completely becomes a carbon precursor [10,23]; the rate of this process is controlled by how readily the acid penetrates the polymer and hence by its crystallinity and hydrophilicity [24]. Our group has recently applied this approach to waste polypropylene and to ABS 3D-printing waste, obtaining carbon anodes with reversible capacities above 300 mA h g^−1^ in lithium half-cells [10,11]. Notably, Villagómez-Salas et al. [13] demonstrated a solvothermal sulfonation approach followed by thermal treatment that successfully converted waste LDPE and HDPE into turbostratic carbon structures with specific capacities of 230 and 350 mA h g^−1^, respectively. However, systematic comparative studies examining LDPE and HDPE derived from actual municipal waste, processed under identical sulfonation and carbonization protocols, remain scarce. The influence of precursor branching and crystallinity on the final carbon’s interlayer spacing, crystallite dimensions, and defect concentration and, by extension, on lithium storage behavior, has not been rigorously evaluated under controlled processing conditions.

In this work, we address these knowledge gaps by conducting a comparative study of carbons derived from waste LDPE and HDPE sourced from municipal solid waste, employing a two-stage sulfonation—carbonization approach. We deliberately adopt identical processing conditions for both precursors so that differences between the final carbons can be related to the precursors. The resulting materials are characterized using a suite of complementary techniques—including SEM, HRTEM, XRD, Raman spectroscopy, X-ray photoelectron spectroscopy (XPS) and N_2_ physisorption—to interrogate their morphology, microstructure, crystallographic parameters, degree of disorder, surface chemistry and porosity. The carbon residues of the pristine and sulfonated precursors are also reported. Their electrochemical performance as LIB anodes is evaluated through cyclic voltammetry, galvanostatic cycling, rate capability tests and electrochemical impedance spectroscopy (EIS), with particular attention to capacity evolution, coulombic efficiency, and the contributions of different potential regions to lithium storage. The comparison is restricted to one LDPE and one HDPE waste item and is intended as a proof of concept; it provides a basis for future studies on multiple and mixed polyethylene waste streams.

## 2. Materials and Methods

### 2.1. Materials Synthesis

Waste LDPE (food packaging film, resin identification code 4) and HDPE (household chemical bottle, resin identification code 2) were collected from municipal solid waste. Each precursor was taken from a single item, each carbon was prepared in a single synthesis batch, and both items were transparent and showed no visible turbidity, which suggests the absence of pigments and opacifying fillers. Labels and caps were removed before processing. The additive content and previous service history of the two items were not determined; the samples are therefore treated as representative waste items rather than as model LDPE and HDPE resins. The materials were rinsed with distilled water, degreased with ethanol, dried at 60 °C for 24 h and cut into fragments of approximately 1 mm × 1 mm. Concentrated sulfuric acid (95–98%, Sigma-Aldrich, St. Louis, MO, USA), N-methyl-2-pyrrolidone (NMP, 99%, Sigma-Aldrich, St. Louis, MO, USA), poly(vinylidene fluoride) (PVDF) (Sigma-Aldrich, St. Louis, MO, USA) and acetylene black (AB, 99.9%, Alfa Aesar, Ward Hill, MA, USA) were used as received.

The synthesis route is shown in Figure 1. LDPE and HDPE (5 g each) were treated with concentrated sulfuric acid (50 mL) at 150 °C for 6 h. The sulfonated products, denoted by LDPE-S and HDPE-S, were filtered, washed to neutral pH and dried at 60 °C for 24 h [13].

LDPE-S and HDPE-S were carbonized in two stages in a tube furnace (BS-2HTF1100E, Bes Saiman Group, Astana, Kazakhstan) under Ar flow (100 mL min^−1^): heating to 350 °C (5 °C min^−1^, 1 h hold), followed by heating to 900 °C (5 °C min^−1^, 2 h hold) and natural cooling under Ar. Both products were milled under identical conditions in a planetary mill (Pulverisette 7, Fritsch GmbH, Idar-Oberstein, Germany, 450 rpm, 1 h) and are denoted LDPE-C-350/900 and HDPE-C-350/900, where the two numbers give the temperatures of the first and second carbonization stages.

### 2.2. Materials Characterization

Surface morphology and internal microstructure were examined by scanning electron microscopy (SEM; JEOL JSM-IT800, JEOL Ltd., Tokyo, Japan, 7 kV) and Cs-corrected high-resolution transmission electron microscopy (HRTEM; JEOL JEM-ARM200F, JEOL Ltd., Tokyo, Japan, 200 kV).

X-ray diffraction (XRD) patterns of the carbons were recorded on a Rigaku MiniFlex diffractometer (Rigaku Corporation, Tokyo, Japan) with Cu Kα radiation (λ = 1.5406 Å) between 10 and 80° 2θ at 5° min^−1^ with a step of 0.05°. The interlayer spacing d_002_ was obtained by applying Bragg’s law to the position of the (002) reflection, and the crystallite dimensions along the c and a axes, L_c_ and L_a_, were estimated from the widths of the (002) and (100) reflections by the Scherrer equation with shape factors of 0.89 and 1.84, respectively [25]. Instrumental broadening was not subtracted; since the measured reflection widths (3.8–5.8° 2*θ*) are much larger than the instrumental width of a laboratory diffractometer, its contribution to the derived dimensions is small and the same for both samples [26]. The full widths at half maximum used in this calculation are reported in Table 2 together with the derived dimensions, so that the two can be assessed independently.

Raman spectra (Horiba LabRam Evolution, HORIBA Scientific, Kyoto, Japan, 532 nm excitation, 1 mW, 10 s exposure, 10 accumulations) were recorded over 500–2000 cm^−1^. After linear baseline correction (OriginPro 2021, OriginLab Corporation, Northampton, MA, USA), the D band (~1350 cm^−1^) and the G band (~1580 cm^−1^) were fitted with pseudo-Voigt profiles, and the integrated intensity (area) ratio A_D_/A_G_ was taken as a measure of structural disorder in the carbon materials [27,28,29].

Thermogravimetric analysis (TGA) was performed on a NETZSCH STA 449 F5 Jupiter simultaneous thermal analyzer (NETZSCH-Gerätebau GmbH, Selb, Germany) under argon at a heating rate of 10 °C min^−1^ from 30 to 1300 °C; the residual mass at 900 °C, the final carbonization temperature used in this work, is reported as the carbon residue. The surface chemistry was studied by XPS on a Nexsa spectrometer (Thermo Scientific, Waltham, MA, USA), and the high-resolution C 1s, O 1s and S 2p spectra were deconvoluted. N_2_ adsorption–desorption isotherms were measured at 77 K on a Micromeritics 3Flex 3500 analyzer (Micromeritics Instrument Corporation, Norcross, GA, USA). The specific surface area was calculated by the Brunauer–Emmett–Teller (BET) method, and the pore size distribution and pore volumes were obtained with the two-dimensional non-local density functional theory (2D-NLDFT) model [30].

### 2.3. Electrode Preparation and Electrochemical Measurements

To evaluate the electrochemical performance of the synthesized carbon materials, electrodes were prepared by combining the carbon, acetylene black and PVDF binder in a mass ratio of 80:10:10. PVDF was first dissolved in NMP using a THINKY ARV-310P planetary centrifugal vacuum mixer (THINKY Corporation, Tokyo, Japan; 1600 rpm, 10 min); the carbon and the conductive additive were then added and homogenized at 1600 rpm for 20 min. The slurry was cast onto copper foil with a doctor blade at a wet thickness of ~200 μm and vacuum-dried at 60 °C for 24 h. The working electrodes were punched into discs of 14 mm in diameter (1.539 cm^2^). The active-material masses of the electrodes shown were 1.568 mg for LDPE-C-350/900 and 1.907 mg for HDPE-C-350/900, corresponding to mass loadings of 1.02 and 1.24 mg cm^−2^. Typical coating thicknesses of electrodes prepared by the same procedure, determined by subtracting the Cu foil thickness (12.3 µm) from the total electrode thickness, were 46.7 and 42.7 µm, corresponding to electrode densities of 0.52 and 0.57 g cm^−3^, respectively.

CR2025 coin-type half-cells were assembled in an argon-filled glovebox (MBRAUN, Garching, Germany; O_2_ and H_2_O below 0.01 ppm) using lithium metal (lithium chips, Nanografi Nano Technology, Ankara, Türkiye) as the counter and reference electrode, a Celgard 2400 separator (Celgard LLC, Charlotte, NC, USA), and 1 M LiPF_6_ in EC:DEC:DMC (1:1:1 *v*/*v*) as the electrolyte (Sigma-Aldrich, St. Louis, MO, USA). Each cell was filled with 120 µL of electrolyte and rested for 12 h before testing. Two parallel cells were assembled and tested for each material; both reproduced the trends reported below, and data from one representative cell of each material are shown.

Galvanostatic cycling was performed on a Neware battery test system (Neware Technology Ltd., Shenzhen, China) between 0.01 and 3.0 V vs. Li^+^/Li at a current density of 100 mA g^−1^, with a 1 min rest between the lithiation and delithiation half-cycles. Rate capability was measured on separately assembled cells at 0.1, 0.2, 0.5, 1 and 2 A g^−1^ (three cycles at each current density), followed by five cycles at 0.1 A g^−1^. Cyclic voltammetry was recorded on a BioLogic VMP-3e potentiostat (BioLogic, Seyssinet-Pariset, France; EC-Lab software V11.61) over the same potential window at a scan rate of 0.1 mV s^−1^. EIS was measured on the same potentiostat after the 3rd, 50th and 100th cycles at 100 mA g^−1^, in the delithiated state at open-circuit potential, over the frequency range from 100 kHz to 10 mHz. The spectra were fitted in EC-Lab ZFit (|Z|^2^ weighting) with the equivalent circuit R_s_ + (R_SEI_‖CPE_SEI_) + (R_ct_‖CPE_dl_) + CPE_W_, and the Warburg coefficient σ was obtained from the slope of Z′ versus ω^−1/2^ between 0.01 and 0.1 Hz [31].

Differential capacity (dQ/dV) curves for the first three cycles were obtained from the galvanostatic charge–discharge data recorded on the Neware system. The dQ/dV values were calculated by the Neware BTS software (version 8.0) from consecutive data points (voltage spacing of about 1–6 mV) and normalized to the active-material mass; no interpolation or smoothing was applied. Lithiation is plotted as negative and delithiation as positive dQ/dV. Because data-point spacing, resampling and smoothing can change the apparent height and number of weak features [32], only peak positions are discussed. Their robustness was verified by interpolating the cumulative capacity onto uniform 5 and 10 mV voltage grids, which reproduced all features at the same potentials. Quantitative estimates of first-cycle processes are based on integrated capacities, which do not depend on these choices.

### 2.4. Use of Generative AI

During the preparation of this work, the authors used ChatGPT (GPT-5, OpenAI) to assist in generating the graphical abstract and schematic illustration of the synthesis procedure (Figure 1). After using this tool, the authors reviewed and edited the content as needed and take full responsibility for the content of the published article.

## 3. Results

### 3.1. Material Characterization

Sulfonation markedly increases the amount of carbon retained on heating (Table 1). Pristine LDPE and HDPE decompose almost completely to volatile products, leaving 1.21 and 0.06 wt.% at 900 °C, in line with the depolymerization of polyolefins [12,23]. After sulfonation the residues rise to 32.63 wt.% for LDPE-S and 8.82 wt.% for HDPE-S, consistent with cross-linking of the chains by the introduced sulfur-containing groups, which stabilizes them against volatilization [10,23]. The 3.7-fold higher residue of LDPE-S is consistent with deeper sulfonation of the branched, less crystalline LDPE, whose amorphous regions are more accessible to the acid than the lamellae of HDPE [24,33]. The low residue of HDPE-S is a practical drawback of the route for this precursor (Section 4). The TGA residues were obtained on milligram samples in a thermobalance and may differ from the yields obtained in the tube furnace.

SEM images of the LDPE- and HDPE-derived carbons are shown in Figure 2. At low magnification (Figure 2a,d), both samples consist of irregularly shaped particles aggregated into dense micron-scale clusters, with no distinguishable morphological difference between the two precursors at this scale. At higher magnification (Figure 2b,e), however, a clear precursor-dependent difference emerges: LDPE-derived carbon exhibits a loosely packed, cloud-like agglomerate morphology composed of poorly defined, fused sub-particles, whereas HDPE-derived carbon shows discrete, well-defined rounded grains packed into a denser, more uniform arrangement. In both cases, the agglomerates are themselves composed of spherical nanoparticles fused together, indicating that the precursor-dependent difference observed at this scale concerns the packing density and definition of these nanoparticles rather than their basic shape.

HRTEM images (Figure 2c,f) reveal a predominantly amorphous, turbostratic structure for both carbons, characterized by short, curved, randomly oriented graphitic fringes without long-range order. Isolated regions of locally ordered, wavy lattice fringes—highlighted by yellow boxes in Figure 2c,f—indicate the presence of small graphitic nanodomains embedded within the disordered matrix [11]. The SAED patterns (insets in Figure 2c,f) show only diffuse rings without discrete spots, consistent with the absence of long-range crystalline order.

Figure 3a shows the XRD patterns of LDPE-C-350/900 and HDPE-C-350/900. Both exhibit two broad reflections centered near 24° and 43° 2θ, assigned to the (002) and (100) reflections of turbostratic carbon. No reflections of crystalline polyethylene remain, confirming complete conversion of both precursors. A weak feature near 34° 2θ in the pattern of LDPE-C-350/900 is close to the noise level and is not assigned to a crystalline phase. The pronounced broadening of both reflections is consistent with the disordered structure observed by HRTEM (Figure 2c,f). The interlayer spacings derived from the (002) positions are 0.365 nm for LDPE-C-350/900 and 0.366 nm for HDPE-C-350/900, both appreciably larger than the 0.336 nm of graphite. The structural parameters are collected in Table 2, which lists the measured peak widths alongside the dimensions derived from them.

**Table 2:** Structural parameters of LDPE-C-350/900 and HDPE-C-350/900 obtained from XRD and Raman analysis [29]. FWHM values are those used in the Scherrer calculation; N = L_c_/d_002_ + 1 is the average number of stacked layers [34]. Values are rounded to the precision supported by the measurements.

Sample	d_002_ (nm)	FWHM (002) (° 2θ)	L_c_ (nm)	FWHM (100) (° 2θ)	L_a_ (nm)	N	A_D_/A_G_
LDPE-C-350/900	0.365	5.80	1.38	3.76	4.6	4.8	1.01
HDPE-C-350/900	0.366	5.84	1.38	4.02	4.3	4.8	1.02

The two carbons are indistinguishable within the resolution of the present measurements. The difference in d_002_ of 0.001 nm corresponds to a shift in the (002) maximum of 0.07° 2θ, i.e., about 1% of the 5.8° width of that reflection. The difference in L_c_ of 0.008 nm corresponds to a difference of 0.04° in the (002) width, some 0.7% of the width itself. Only L_a_ shows a larger relative difference, about 7%, arising from a difference of 0.26° in the width of the (100) reflection—the weaker and less well-defined of the two features in Figure 3a. Differences of this magnitude, obtained from reflections several degrees wide, do not establish a structural distinction between the samples, and none is claimed here. The average number of stacked layers, N = 4.8, is the same for both carbons.

The Raman spectra (Figure 3b) display the characteristic D band near 1350 cm^−1^ and G band near 1580 cm^−1^, arising from defect-activated breathing modes of aromatic rings and from in-plane sp^2^ stretching, respectively. The two bands are well-resolved, and the intensity between them returns close to the baseline. The integrated intensity ratios A_D_/A_G_, 1.01 for LDPE-C-350/900 and 1.02 for HDPE-C-350/900, differ by 1% and indicate the same high level of structural disorder, in agreement with the diffraction and microscopy results. The bands of LDPE-C-350/900 are somewhat narrower than those of HDPE-C-350/900, which may reflect a narrower distribution of local structural environments; because the area ratio is the same for both carbons, this difference is not taken as evidence of a different degree of graphitic order.

The surface composition of the carbons was examined by XPS (Figure 4). The survey spectra (Figure 4a) show only C, O and S. Both carbons consist predominantly of carbon (94.71 and 91.65 at.% for LDPE-C-350/900 and HDPE-C-350/900), with 4.78 and 8.01 at.% O and only 0.51 and 0.34 at.% S (Figure 4b). The C 1s spectra (Figure 4c,f) are dominated by the sp^2^ C=C/C–C component at 284.8 eV, accompanied by minor C–S, C–O–C and O=C–O components. Each S 2p spectrum (Figure 4d,g) contains two spin–orbit doublets, one at ≈164–165 eV assigned to thiophene-type C–S–C sulfur and one at ≈168–171 eV assigned to oxidized C–SO_x_–C sulfur [11,35], while the O 1s spectra (Figure 4e,h) comprise C=O, C–O and S=O contributions. Most of the sulfur introduced by sulfonation is therefore removed during carbonization at 900 °C, and the small fraction that remains is covalently bound in the carbon framework, in agreement with sulfonated polypropylene carbonized at the same temperature (≤0.2 at.% S) [10].

The N_2_ physisorption isotherms of both carbons (Figure 5a) show low uptake at low relative pressure, a steep rise above P/P_0_ ≈ 0.8 and a narrow hysteresis loop, characteristic of materials whose porosity is dominated by mesopores and interparticle voids rather than micropores [30]. LDPE-C-350/900 has a larger BET surface area than HDPE-C-350/900 (53.7 vs. 32.5 m^2^ g^−1^) and a larger total pore volume (0.183 vs. 0.135 cm^3^ g^−1^) (Table 3). In both carbons micropores contribute little: the external surface area is practically equal to the BET area, and mesopores account for about 95% of the 2D-NLDFT pore volume (Figure 5b–d). The pores of LDPE-C-350/900 are also somewhat narrower (average width 13.6 vs. 16.6 nm), with a pore-size distribution peaking near 15–20 nm rather than 25–35 nm (Figure 5c). The two carbons therefore differ in accessible surface and pore volume while sharing the same bulk turbostratic structure. This difference agrees with the looser, finer agglomerates of LDPE-C-350/900 observed by SEM (Figure 2b) and with the higher carbon residue of LDPE-S (Table 1).

### 3.2. Electrochemical Characterization

Cyclic voltammograms recorded between 0.01 and 3.0 V vs. Li^+^/Li at 0.1 mV s^−1^ are shown in Figure 6a,b. In the first cathodic scan (lithiation) both electrodes pass an irreversible current between approximately 1.0 and 0.3 V that is absent from the subsequent scans. In HDPE-C-350/900 this contribution appears as a distinct peak near 0.7 V (Figure 6b), whereas in LDPE-C-350/900 it is distributed over a wider potential interval without a resolved maximum (Figure 6a). The distinction concerns the distribution of the irreversible process over potential and not its magnitude, since the total first-cycle irreversible capacity is in fact the larger for LDPE-C-350/900, as shown below. Beyond the first scan the irreversible features are absent and the voltammograms of both electrodes retain the same shape, indicating that the interfacial process is essentially complete after one cycle. The cathodic current and the anodic delithiation peak near 0.3 V nevertheless continue to grow from the second to the third cycle, in agreement with the progressive increase in capacity observed in galvanostatic cycling and discussed below.

Galvanostatic charge–discharge profiles for the 1st, 2nd, 3rd, 50th and 100th cycles at 100 mA g^−1^ are presented in Figure 7a,b. In the first cycle LDPE-C-350/900 delivered a lithiation capacity of 377.5 mA h g^−1^ and a delithiation capacity of 209.9 mA h g^−1^, corresponding to an initial coulombic efficiency (ICE) of 55.6%, while HDPE-C-350/900 delivered 297.3 and 187.7 mA h g^−1^, giving an ICE of 63.1%. The first-cycle irreversible capacities are therefore 167.6 and 109.6 mA h g^−1^.

The electrodes compared here differ in active-material loading by about 22% (1.02 vs. 1.24 mg cm^−2^). At such low loadings and coating thicknesses below 50 µm, ion transport through the electrode is not expected to limit utilization at 100 mA g^−1^ [8,36], and first-cycle losses of carbon electrodes scale primarily with the surface area exposed to the electrolyte rather than with the electrode mass [37]. A small contribution of the lower loading of the LDPE-C-350/900 electrode to its higher utilization cannot be excluded; however, the loading difference is much smaller than the 65% difference in BET surface area (Table 3), and the capacity ordering is reproduced in the parallel cells and in the separately assembled rate-test cells (Table 4).

The differential capacity (dQ/dV) curves derived from the first three galvanostatic cycles (Figure 7c,d) resolve the first-cycle irreversible process against potential. In all cycles the lithiation signal of both electrodes rises steeply towards the 0.01 V cut-off without a resolved maximum, whereas the main delithiation peak grows from the first to the second and third cycles and shifts from 0.15 to 0.11 and 0.10 V for LDPE-C-350/900 and from 0.20 to 0.12 and 0.11 V for HDPE-C-350/900, consistent with the growth of the reversible peaks noted in the CV data. Superimposed on this trend, the first cycle of each electrode carries additional features, absent from later cycles and not resolved by CV alone. LDPE-C-350/900 shows one pronounced feature on its lithiation branch at 0.32 V and two weaker, narrow features at 0.44 and 0.63 V. HDPE-C-350/900, by contrast, shows a single broad feature centered at 0.62 V, matching the resolved 0.7 V peak seen by CV for this electrode (Figure 6b), together with a small counterpart on the delithiation branch near 1.0 V that weakens in the second cycle and is no longer resolved by the third. All of these features remained at the same potentials when the data were resampled on uniform voltage grids (Section 2.3); their heights, however, depend on the data-point spacing and are therefore not compared quantitatively. This asymmetry—a distributed first-cycle response for LDPE-C-350/900 against one localized feature for HDPE-C-350/900—parallels the CV data.

The first-cycle irreversible capacity of disordered carbons comprises electrolyte reduction to form the solid electrolyte interphase (SEI) and irreversible trapping of Li^+^ at defects, pores and heteroatom sites [38,39]. To estimate these contributions, the irreversible capacity was partitioned with the help of the first two cycles into (i) the excess of first-cycle over second-cycle lithiation charge above 0.25 V, where the carbonate electrolyte is reduced [40]; (ii) the corresponding excess below 0.25 V; and (iii) the difference between the second-cycle lithiation and the first-cycle delithiation capacities, i.e., lithium inserted in the first cycle but not extracted. For HDPE-C-350/900 these terms amount to 46, ≈0 and 64 mA h g^−1^ (43, 0 and 57% of the irreversible capacity), and for LDPE-C-350/900 to 47, 58 and 63 mA h g^−1^ (28, 35 and 38%). The charge consumed above 0.25 V, which we assign to SEI formation, is therefore nearly the same for both electrodes and differs only in its distribution over potential, and the amount of lithium retained after the first cycle is also similar. The entire additional irreversible capacity of LDPE-C-350/900 is consumed below 0.25 V in the first cycle, where continued SEI growth and Li^+^ trapping in pores cannot be separated. Because this contribution appears only for the carbon with the 65% larger BET area and 36% larger pore volume (Table 3), and the irreversible capacity normalized to the BET area is similar for both carbons (3.1 and 3.4 mA h m^−2^), we relate the larger first-cycle loss of LDPE-C-350/900 to its larger electrolyte-accessible surface [37]. This partition is approximate, because the processes overlap in potential and the second-cycle capacity already contains a contribution from activation.

Both electrodes deliver capacity across the whole potential window without a flat plateau, but the slope of the profile changes markedly at low potential. At the 100th cycle, 28% of the lithiation capacity of LDPE-C-350/900 and 24% of that of HDPE-C-350/900 is delivered below 0.1 V, that is 95 and 74 mA h g^−1^ within an interval only 0.09 V wide, against 90 and 79 mA h g^−1^ across the three times wider interval from 0.5 to 0.2 V.

Coulombic efficiency rose rapidly over the first ten cycles, reaching 98.3% for LDPE-C-350/900 and 98.8% for HDPE-C-350/900, and averaged 99.4% for both electrodes over cycles 50 to 100 (Figure 7e,f). The reversible delithiation capacity increased progressively with cycling, from 258.9 to 312.1 and 337.5 mA h g^−1^ at cycles 3, 50 and 100 for LDPE-C-350/900, and from 249.5 to 285.7 and 302.2 mA h g^−1^ for HDPE-C-350/900.

Between cycles 2 and 100 the capacity delivered above 1.0 V vs. Li^+^/Li—above the interval in which the bulk of the lithium capacity of these carbons is delivered, and within the interval in which reduction in the carbonate electrolyte proceeds [40]—rose from 7.5 to 18.4 mA h g^−1^ for LDPE-C-350/900 and from 14.1 to 24.1 mA h g^−1^ for HDPE-C-350/900, accounting respectively for 17% and 20% of the total capacity gain over that interval. Over the same range of cycles, the fraction of capacity delivered below 0.1 V remained essentially constant, at about 28–31% for LDPE-C-350/900 and 24–25% for HDPE-C-350/900. Over cycles 50 to 100 the steady-state coulombic efficiency of 99.4% corresponds to a cumulative irreversible charge of 94.9 mA h g^−1^ for LDPE-C-350/900 and 91.2 mA h g^−1^ for HDPE-C-350/900, some 3.7 and 5.5 times the capacity gained over the same interval, 25.4 and 16.5 mA h g^−1^. Overall, the two carbons are not equivalent in capacity, and the difference widens with cycling: the reversible capacity of LDPE-C-350/900 exceeds that of HDPE-C-350/900 by 3.8% at cycle 3, by 9.2% at cycle 50 and by 11.7% at cycle 100.

The rate capability is shown in Figure 8 and summarized in Table 4. As the current density increases from 0.1 to 2 A g^−1^, the capacity decreases from 249.6 to 154.5 mA h g^−1^ for LDPE-C-350/900 and from 241.2 to 130.1 mA h g^−1^ for HDPE-C-350/900, corresponding to retentions of 61.9 and 54.0%. LDPE-C-350/900 delivers the higher capacity at every current density, and the difference grows from 3.5% at 0.1 A g^−1^ to 18.8% at 2 A g^−1^. The charge–discharge profiles (Figure 8c,d) show increasing polarization and a shrinking low-potential contribution as the current rises, as expected when Li^+^ diffusion into the carbon becomes rate-limiting [10]. On return to 0.1 A g^−1^ both electrodes recover more than their initial capacity (290.9 and 266.8 mA h g^−1^, i.e., 116.5 and 110.6%), which shows that high-rate operation does not damage the carbon framework [41] and that the activation observed in prolonged cycling continues during the rate test, as also reported for sulfonated polypropylene-derived carbons [10].

EIS spectra recorded after the 3rd, 50th and 100th cycles (Figure 9) consist of a high-frequency intercept, two partially overlapping depressed semicircles and a low-frequency sloping line. They were fitted with the equivalent circuit in Figure 9e, in which the high-frequency arc is assigned to Li^+^ migration through the SEI (R_SEI_, CPE_SEI_), the mid-frequency arc to charge transfer (R_ct_, CPE_dl_) and the low-frequency line to solid-state diffusion; constant phase elements (CPEs) account for the non-ideal response of the porous electrodes [31,42]. The fitted parameters are listed in Table 5. After the 3rd cycle the interfacial resistance R_SEI_ + R_ct_ is similar for the two electrodes (48.1 and 51.5 Ω) but is distributed differently: HDPE-C-350/900 has the larger R_SEI_ (48.0 vs. 35.0 Ω) and a much smaller R_ct_ (3.5 vs. 13.1 Ω). During cycling R_SEI_ decreases by a factor of two for LDPE-C-350/900 and of about four for HDPE-C-350/900, to 17.6 and 12.4 Ω after 100 cycles, and the Warburg coefficient σ falls from 24.7 to 14.5 Ω s^−1/2^ and from 23.9 to 15.9 Ω s^−1/2^, respectively. A decrease in film resistance and diffusion impedance with cycling, rather than the increase expected for a thickening passivation layer, indicates progressive wetting and conditioning of the electrode, which enlarges the electrochemically active interface and shortens the effective diffusion paths [7,43]. These trends coincide with the rise in reversible capacity (Figure 7e,f) and support, although they do not prove, the interpretation of the capacity gain as gradual electrochemical activation. At the 100th cycle the total interfacial resistance is lower for HDPE-C-350/900 (15.5 vs. 25.6 Ω), although this electrode delivers the lower capacity; the capacity difference between the two carbons is therefore unlikely to be governed by interfacial kinetics.

## 4. Discussion

The structural results in Section 3.1 show that LDPE-C-350/900 and HDPE-C-350/900 are indistinguishable within the resolving power of XRD and HRTEM and have the same Raman disorder ratio: the interlayer spacing, crystallite dimensions, stacking number and disorder ratio of the two carbons differ by margins smaller than the measurement uncertainty of each technique. This bulk similarity coexists with a clear morphological distinction at the SEM scale, where LDPE-derived carbon forms a loosely packed, cloud-like agglomerate and HDPE-derived carbon forms discrete, more uniformly packed grains, and with a 65% larger surface area of the LDPE-derived carbon (Table 3). A plausible origin for this combination lies in the precursor-dependent kinetics of the sulfonation step rather than in the subsequent carbonization, which is common to both precursors. Sulfonation and cross-linking of polyolefins have been reported to depend primarily on precursor crystallinity, hydrophilicity and on the availability of tertiary carbon sites rather than on other processing parameters [24,44], and the tightly packed, linear chains characteristic of HDPE have been shown to restrict acid penetration and hinder complete aromatization relative to branched structures [33]. On this basis, the more branched, less crystalline LDPE would be expected to permit more extensive and more spatially distributed acid diffusion and cross-linking during sulfonation than the linear, more crystalline HDPE, producing the looser agglomerate morphology observed by SEM while leaving the graphitic domain structure set by carbonization essentially unchanged. The almost four times higher carbon residue of LDPE-S (Table 1), which reflects the extent of cross-linking introduced by sulfonation [23], supports this picture. This account is consistent with reports that sulfonation–carbonization routes for polyolefins converge on a common turbostratic microstructure largely independent of the specific PE grade [45]. The branching, crystallinity and additive content of the two waste items and the sulfur content of the sulfonated intermediates were, however, not measured directly; the sulfonation-based account is therefore offered as the interpretation most consistent with the present data rather than as an established mechanism.

The electrochemical data are consistent with the same precursor-dependent asymmetry. The first-cycle CV and dQ/dV data resolve a single, well-defined irreversible feature for HDPE-C-350/900, centered near 0.6–0.7 V, against one pronounced and two weaker features for LDPE-C-350/900 spanning roughly 0.3–0.6 V. This pattern parallels the SEM contrast: a more confined, spatially uniform sulfonation response in HDPE would be expected to generate a more homogeneous population of reactive sites and, correspondingly, a single dominant interfacial process at a well-defined potential, whereas the more extensive and less uniform acid penetration inferred for LDPE would generate a wider range of local chemical environments and, correspondingly, several distinguishable interfacial processes at different potentials.

The first-cycle irreversible capacities of 167.6 and 109.6 mA h g^−1^ for LDPE-C-350/900 and HDPE-C-350/900, respectively, are consistent with the combined contribution of SEI formation on the exposed carbon surface and irreversible Li^+^ trapping at defect sites, both expected to be significant in disordered carbons of this kind [39,40,46]. Values in this range are typical of disordered carbons cycled against lithium, where a high density of defects and accessible surface area promotes extensive interphase formation and depresses the initial coulombic efficiency relative to graphite [39,46]. The N_2_ physisorption data point to a common origin for the larger first-cycle loss and the higher reversible capacity of LDPE-C-350/900: its 65% larger surface area and 36% larger pore volume. The SEI-related charge consumed above 0.25 V is nearly the same for both electrodes, the additional first-cycle loss of LDPE-C-350/900 appears below 0.25 V, and the irreversible capacity normalized to the BET area is similar for both carbons (Section 3.2), as expected when first-cycle losses scale with the electrolyte-accessible surface [37]; at the same time the larger mesopore volume and finer agglomerates provide more sites for Li^+^ adsorption and shorter diffusion paths [11,38], consistent with the higher capacity of LDPE-C-350/900 at every current density (Table 4).

Residual heteroatoms are an alternative explanation for the capacity difference, because sulfur and oxygen functional groups can add capacity, trap Li^+^ irreversibly and modify the SEI [11,38]. The XPS data make a sulfur-based explanation unlikely. The two carbons retain only 0.51 and 0.34 at.% S, and even if the entire 0.17 at.% excess of sulfur in LDPE-C-350/900 reacted reversibly with two Li^+^ per S atom, it would contribute about 7–8 mA h g^−1^, less than a quarter of the 35 mA h g^−1^ difference at cycle 100. Oxygen, in turn, is more abundant in HDPE-C-350/900 (8.01 vs. 4.78 at.%), the carbon with the lower capacity and the lower first-cycle loss. These estimates refer to the near-surface region probed by XPS, but together they indicate that residual heteroatoms are not the main source of the difference between the two carbons; as for sulfonated polypropylene-derived carbon, the residual sulfur content is too low to contribute appreciably [10].

The progressive increase in reversible capacity observed over 100 cycles for both electrodes is tentatively attributed to gradual electrochemical activation. The capacity delivered above 1.0 V vs. Li^+^/Li—within the potential range associated with carbonate electrolyte reduction [40]—increases in absolute terms over cycling, while the fraction delivered below 0.1 V remains roughly constant. Together with the decrease in R_SEI_ and σ (Table 5) and the recovery of more than 100% of the initial capacity after the rate test (Table 4), this pattern is consistent with progressive electrolyte wetting of pores and surfaces that are initially inaccessible, as reported for porous carbons [10,47]. Continued evolution of the SEI and slow rearrangement of the disordered carbon framework may also contribute [38,43], and the present measurements do not identify the mechanism directly. The average coulombic efficiency of 99.4% over cycles 50–100 also shows that side reactions continue: the corresponding cumulative irreversible charge (94.9 and 91.2 mA h g^−1^) exceeds the capacity gained over the same interval, so the electrodes are still evolving at cycle 100 rather than cycling stably.

Because the capacity is still rising at cycle 100, the long-term behavior cannot be extrapolated with confidence. For polypropylene-derived carbon prepared by the same route, a period of activation was followed by gradual fading [10], and continued electrolyte consumption at a coulombic efficiency of 99.4% would limit the life of full cells with a finite lithium inventory [38]. The capacity is therefore expected to level off and eventually decline on prolonged cycling, and extended cycling over several hundred cycles is required to establish the cycle life of these materials.

Table 6 compares the present carbons with carbons from other waste feedstocks and with typical hard carbons. Their reversible capacities after 100 cycles (302–338 mA h g^−1^) are comparable with those of carbons from sulfonated polypropylene packaging (373 mA h g^−1^) and cross-linked ABS waste (>300 mA h g^−1^) [10,11], higher than that of activated carbon from olive pomace measured at a lower current density [9], and within the range reported for LDPE- and HDPE-derived carbons by Villagómez-Salas et al. [13]. Their capacity retention at 2 A g^−1^ (54–62%) lies within the range reported for sulfonated polypropylene-derived carbon (38–65%, depending on carbonization temperature) [10]. The initial coulombic efficiencies (55.6 and 63.1%) are higher than those of carbons from polypropylene packaging and disposable masks (30–48%) [10,48] but lie at the lower end of the 50–80% typical of hard carbons [38], and the reversible capacities remain below the theoretical capacity of graphite (372 mA h g^−1^) [38]. The low ICE is the main practical limitation of these materials; it could be addressed by reducing the accessible surface area, carbon coating, film-forming electrolyte additives or pre-lithiation [38].

The present comparison was carried out on carbons derived from one LDPE food packaging film and one HDPE household chemical bottle, processed under an identical sulfonation and carbonization protocol. Under these conditions, two polyethylene grades of nominally different structure yield carbons of indistinguishable bulk crystallographic structure and Raman disorder ratio, but different carbon residue after sulfonation, surface area, first-cycle interfacial response and capacity. These observations are consistent with the precursor influencing the final anode through the sulfonation step rather than through carbonization itself. Whether this holds generally, and whether the route tolerates mixed polyethylene streams, remains to be established with multiple waste sources, mixed feeds and practically relevant electrode loadings, at which ion transport through thick electrodes imposes additional constraints [7,8].

## 5. Conclusions

Waste LDPE and HDPE, processed under identical sulfonation and carbonization conditions, give carbons that are indistinguishable within the resolution of XRD and HRTEM, with the same Raman disorder ratio, despite the nominally different branching, density and crystallinity of the two polyethylene grades. Sulfonation raises the carbon residue at 900 °C to 32.63 wt.% for LDPE and 8.82 wt.% for HDPE, consistent with deeper sulfonation of the more accessible amorphous structure of LDPE; little sulfur remains in either carbon (≤0.51 at.%). The LDPE-derived carbon has a larger surface area (53.7 vs. 32.5 m^2^ g^−1^), a larger first-cycle irreversible capacity, a broader potential distribution of first-cycle interfacial processes and a higher reversible capacity: 338 vs. 302 mA h g^−1^ after 100 cycles at 100 mA g^−1^, and 155 vs. 130 mA h g^−1^ at 2 A g^−1^. In both electrodes the capacity is still increasing at cycle 100, accompanied by decreasing SEI and diffusion impedance, which points to gradual activation; long-term stability remains to be established. As a proof of concept, these results show that sulfonation-assisted carbonization converts both polyethylene grades into anode-active disordered carbons, with the differences between them consistent with the precursor acting mainly through its response to sulfonation. Testing of multiple and mixed waste streams and of practically relevant electrode loadings, together with improvement of the initial coulombic efficiency, are the next steps toward practical use.

## Figures and Tables

**Figure 1:**
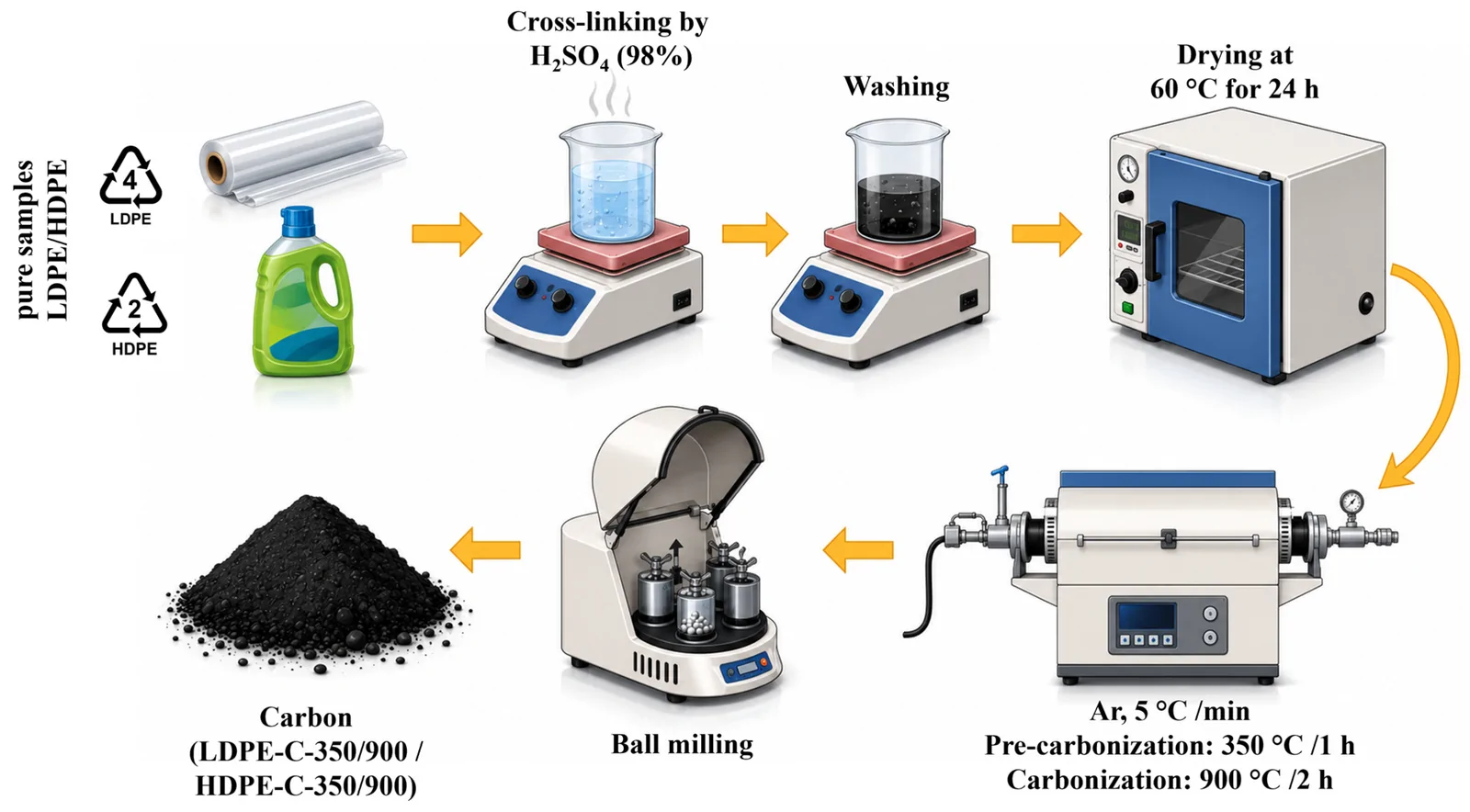
Schematic illustration of the synthesis route for carbon materials derived from waste polyethylene.

**Figure 2:**
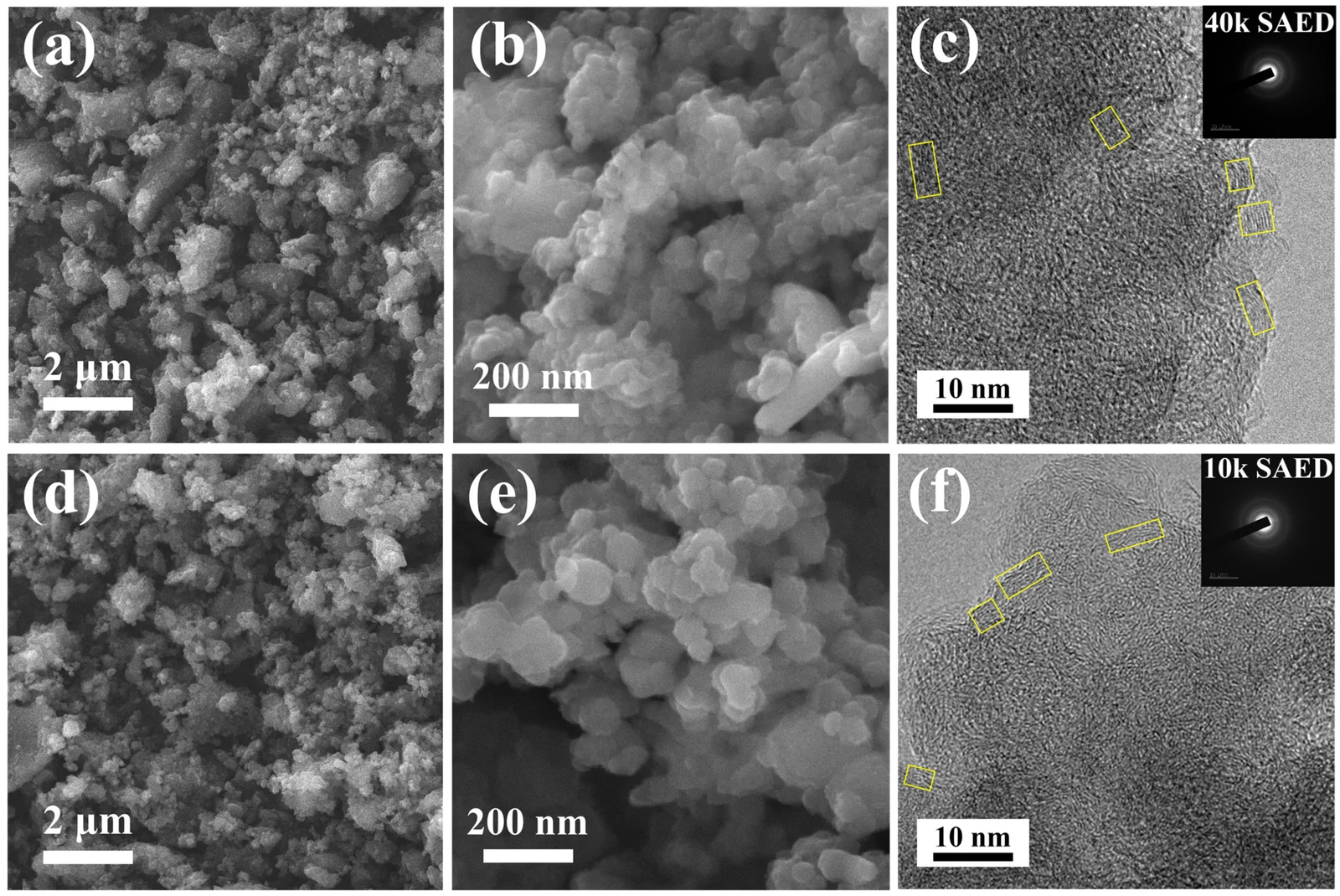
Morphological and microstructural characterization of carbon materials derived from waste LDPE and HDPE: SEM images of LDPE-derived carbon at (**a**) low and (**b**) high magnification, (**c**) HRTEM image; SEM images of HDPE-derived carbon at (**d**) low and (**e**) high magnification, (**f**) HRTEM image; insets in (**c**,**f**): selected-area electron diffraction (SAED) patterns; yellow boxes in (**c**,**f**) mark regions of locally ordered graphitic fringes.

**Figure 3:**
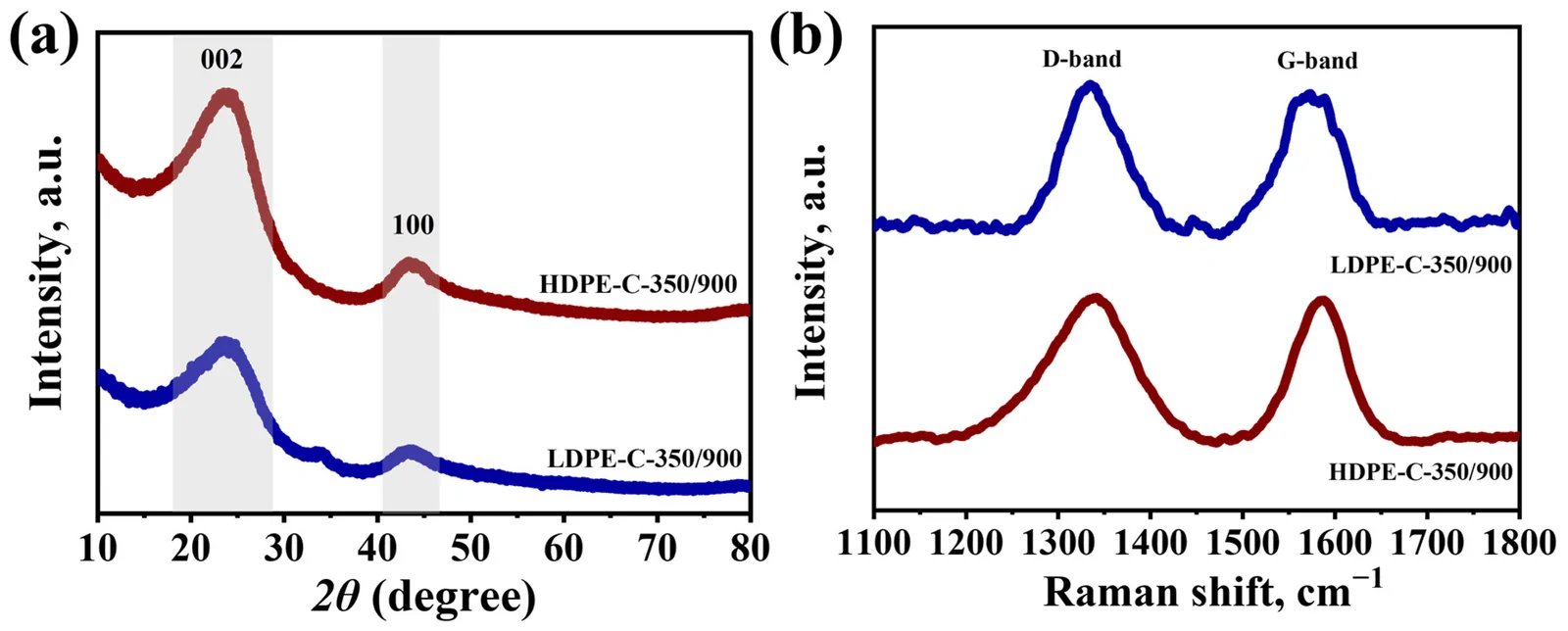
(**a**) X-ray diffraction patterns and (**b**) Raman spectra of carbon materials derived from waste LDPE and HDPE. Gray bands in (**a**) mark the regions of the (002) and (100) reflections.

**Figure 4:**
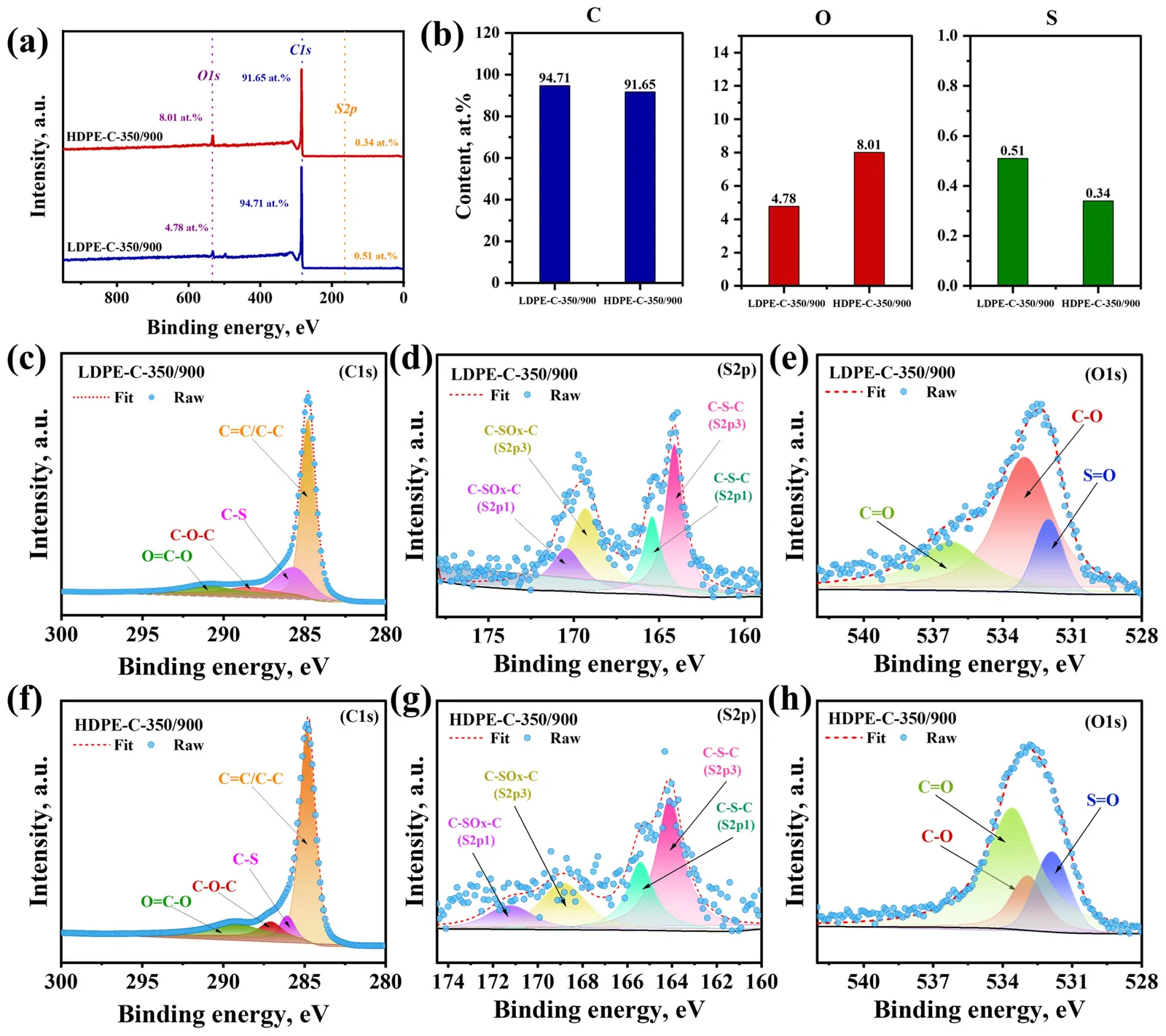
XPS characterization of LDPE-C-350/900 and HDPE-C-350/900: (**a**) survey spectra; (**b**) surface elemental composition (C, O and S, at.%); high-resolution deconvoluted C 1s (**c**,**f**), S 2p (**d**,**g**) and O 1s (**e**,**h**) spectra of LDPE-C-350/900 (**c**–**e**) and HDPE-C-350/900 (**f**–**h**).

**Figure 5:**
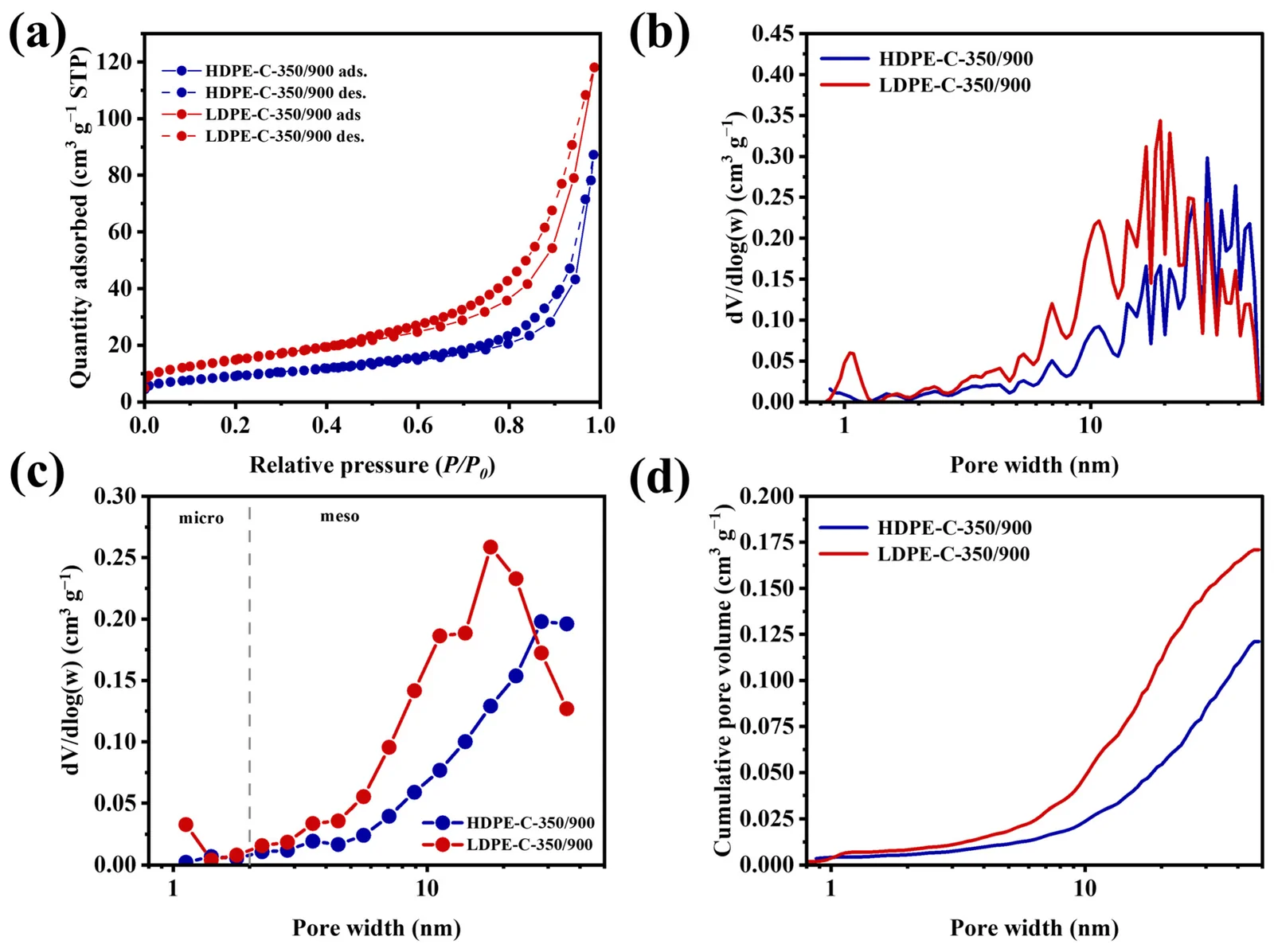
N_2_ physisorption analysis of LDPE-C-350/900 and HDPE-C-350/900 at 77 K: (**a**) N_2_ adsorption–desorption isotherms; (**b**) 2D-NLDFT pore size distributions; (**c**) 2D-NLDFT pore size distributions recalculated on a logarithmic grid, with the dashed line marking the micropore/mesopore boundary (2 nm); (**d**) cumulative pore volume as a function of pore width.

**Figure 6:**
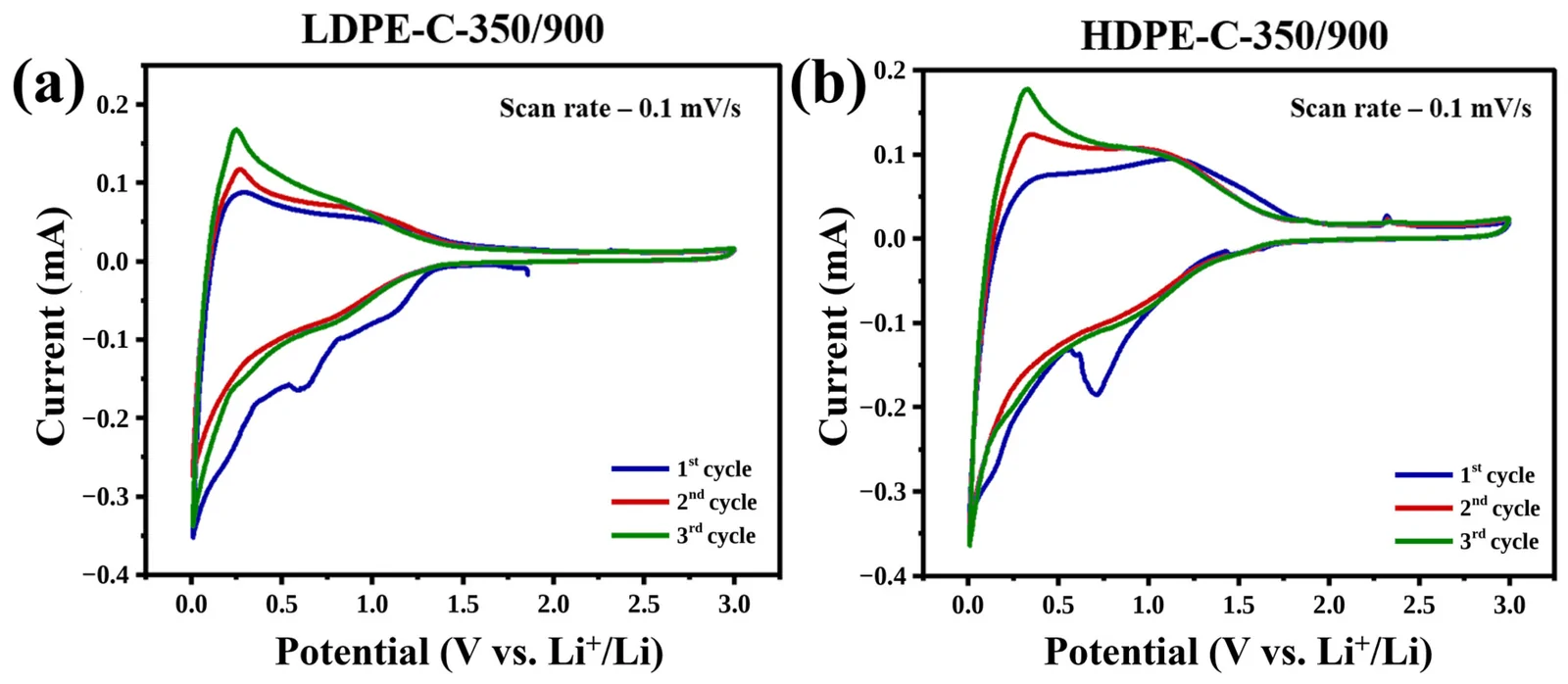
Cyclic voltammograms of (**a**) LDPE-C-350/900 and (**b**) HDPE-C-350/900 for the first three cycles, recorded at 0.1 mV s^−1^ between 0.01 and 3.0 V vs. Li^+^/Li.

**Figure 7:**
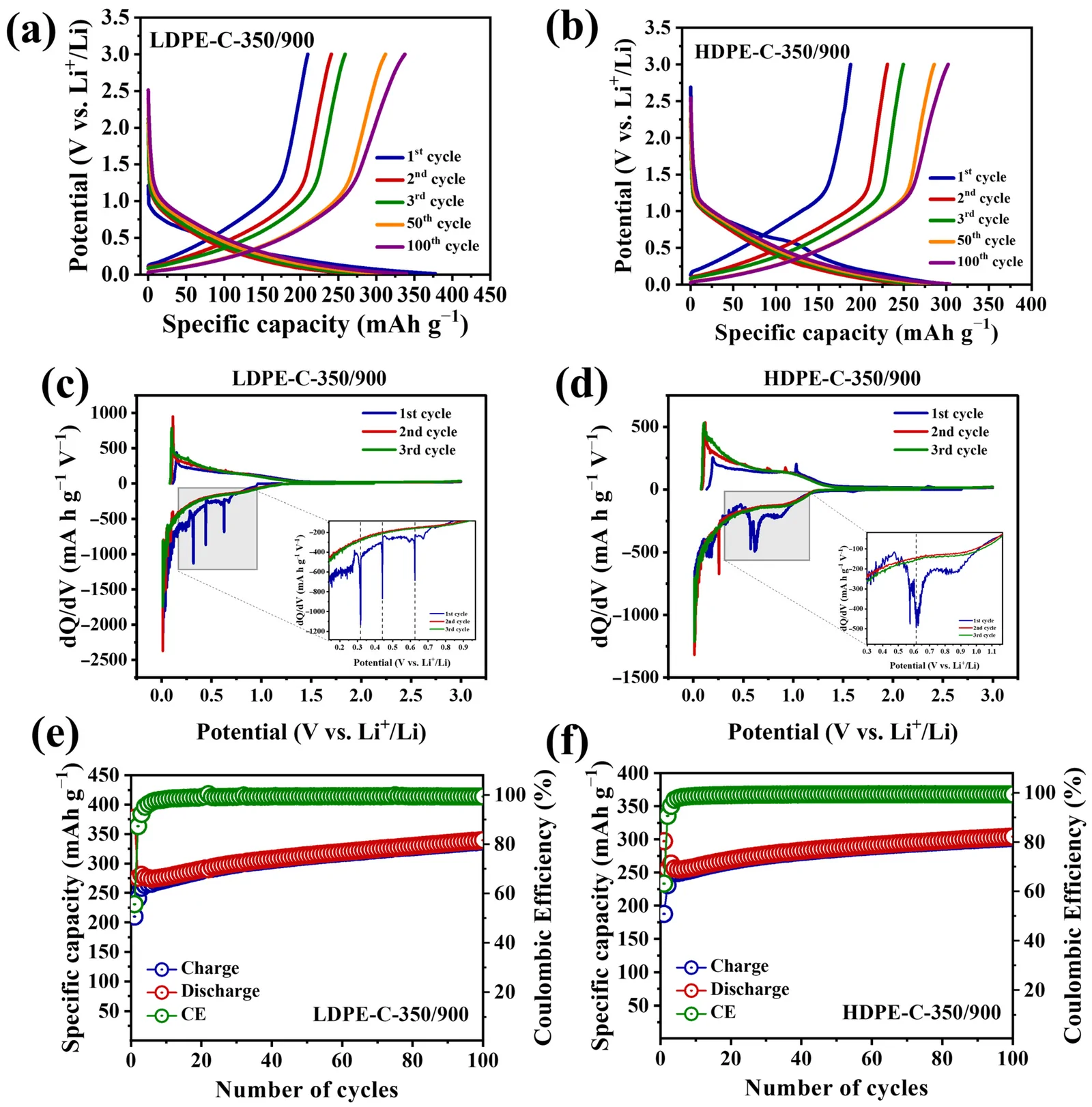
Electrochemical performance of the carbon materials: galvanostatic charge–discharge profiles of (**a**) LDPE-C-350/900 and (**b**) HDPE-C-350/900 for the 1st, 2nd, 3rd, 50th and 100th cycles; differential capacity (dQ/dV) curves of (**c**) LDPE-C-350/900 and (**d**) HDPE-C-350/900 for the first three cycles; cycling performance and coulombic efficiency of (**e**) LDPE-C-350/900 and (**f**) HDPE-C-350/900.

**Figure 8:**
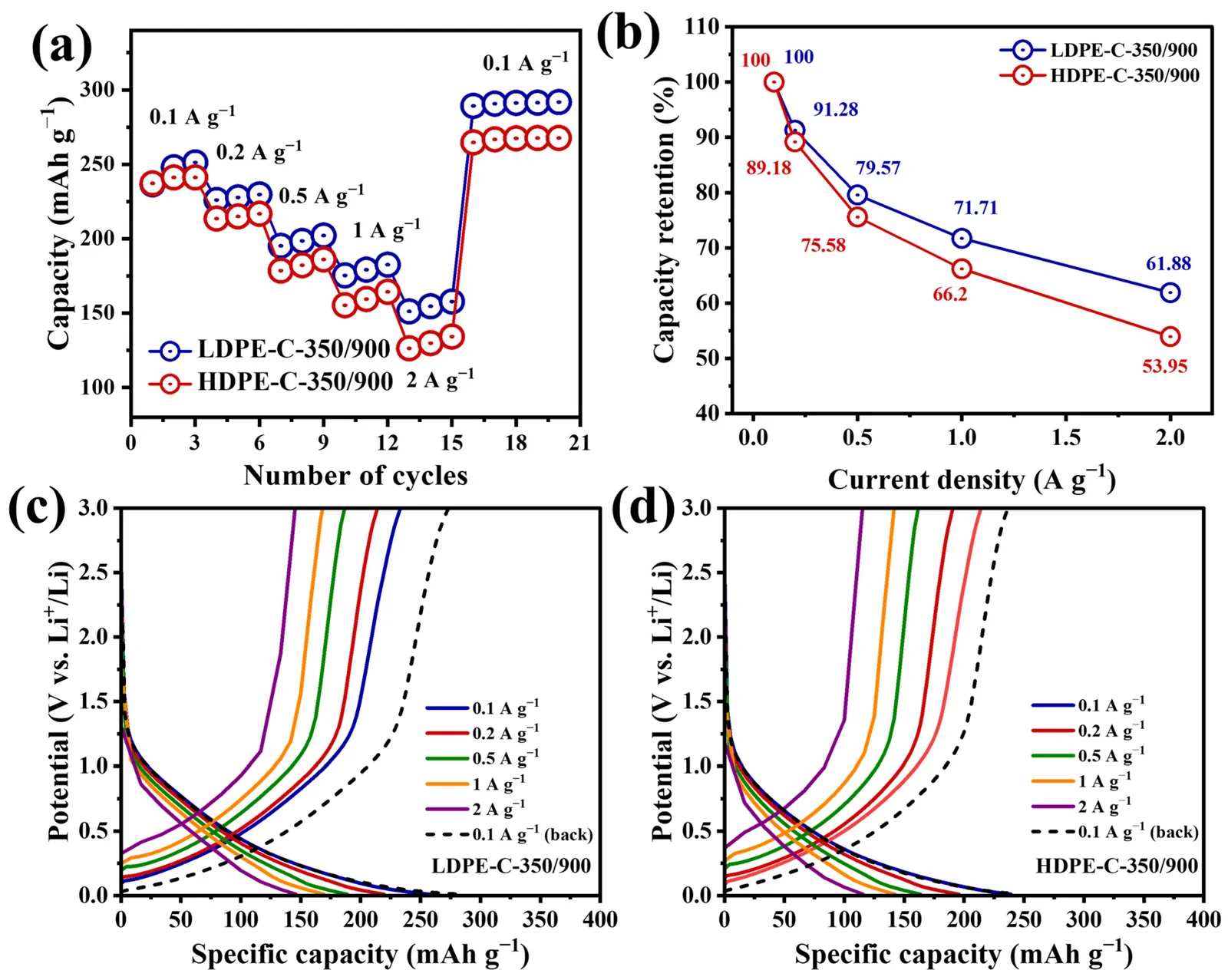
Rate performance of LDPE-C-350/900 and HDPE-C-350/900 anodes: (**a**) rate capability at 0.1–2 A g^−1^; (**b**) capacity retention vs. current density; (**c**,**d**) charge–discharge profiles at different current densities.

**Figure 9:**
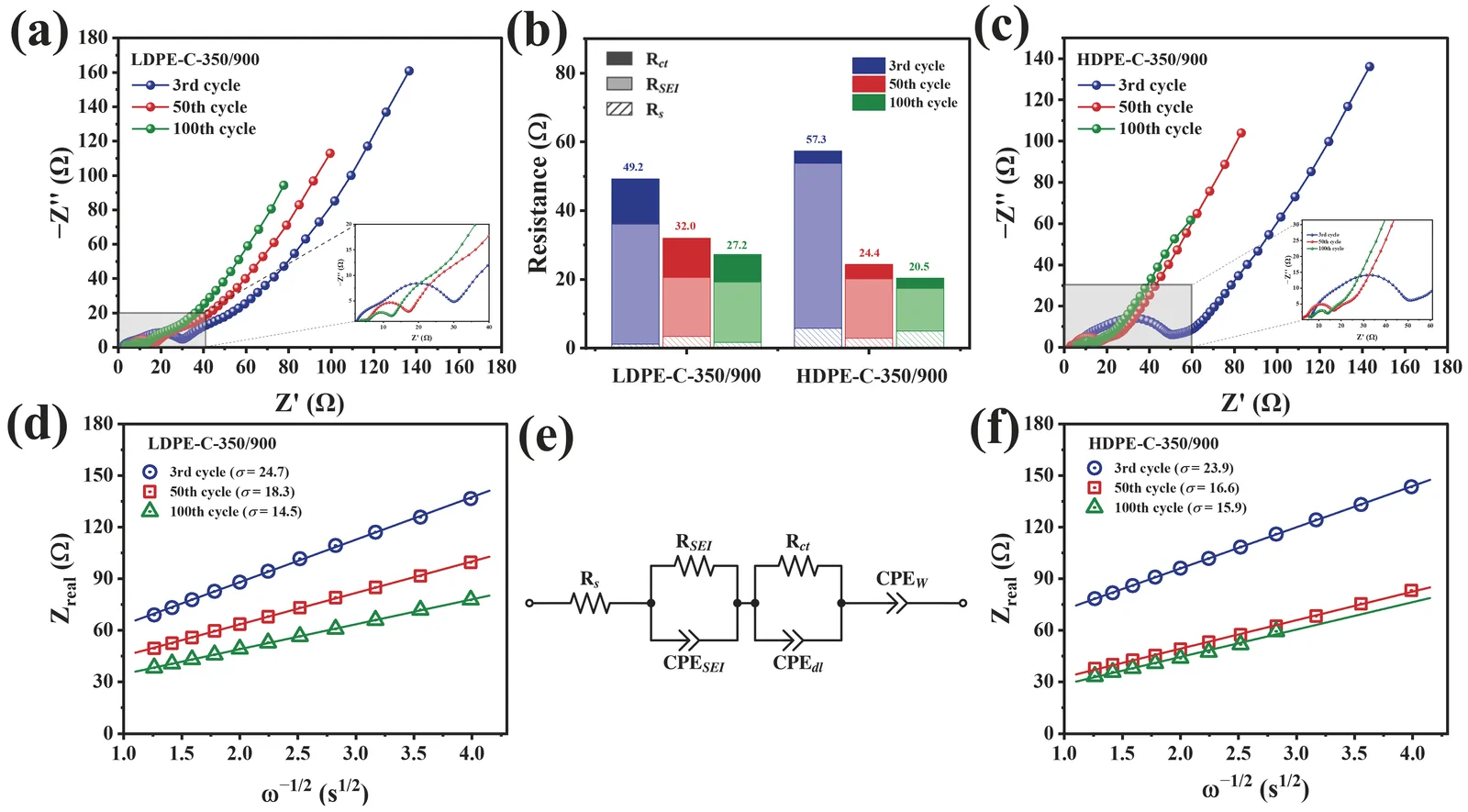
EIS of LDPE-C-350/900 and HDPE-C-350/900 after 3, 50 and 100 cycles: (**a**,**c**) Nyquist plots; (**b**) fitted R_s_, R_SEI_ and R_ct_; (**d**,**f**) Z_real_ vs. ω^−1/2^ at low frequencies; (**e**) equivalent circuit.

**Table 1:** Carbon residue at 900 °C of the pristine and sulfonated precursors (TGA, Ar, 10 °C min^−1^).

Sample	Residue at 900 °C (wt.%)
LDPE	1.21
LDPE-S	32.63
HDPE	0.06
HDPE-S	8.82

**Table 3:** Textural parameters of LDPE-C-350/900 and HDPE-C-350/900 derived from N_2_ physisorption at 77 K. V_total_ is the single-point pore volume at P/P_0_ ≈ 0.99; V_meso_ and V_DFT_ are the 2D-NLDFT pore volumes in pores of 2–50 nm and in all pores, respectively.

Sample	S_BET_ (m^2^ g^−1^)	C_BET_	S_ext_ (m^2^ g^−1^)	V_total_ (cm^3^ g^−1^)	V_micro_ (cm^3^ g^−1^)	V_meso_ (cm^3^ g^−1^)	V_meso_/V_DFT_ (%)	D_avg_ (nm)
LDPE-C-350/900	53.7 ± 0.1	95	53.8	0.183	0.019	0.169	95.4	13.6
HDPE-C-350/900	32.5 ± 0.1	118	32.2	0.135	0.011	0.123	95.7	16.6

**Table 4:** Rate performance of LDPE-C-350/900 and HDPE-C-350/900 anodes. Capacities are averaged charge (delithiation) capacities over the cycles at each current density; at the initial 0.1 A g^−1^ step the first (formation) cycle is excluded.

Current Density (A g^−1^)	C-Rate *	LDPE-C-350/900 Capacity (mA h g^−1^)	LDPE-C-350/900 Retention (%)	HDPE-C-350/900 Capacity (mA h g^−1^)	HDPE-C-350/900 Retention (%)
0.1	0.27C	249.6	100	241.2	100
0.2	0.54C	227.8	91.3	215.1	89.2
0.5	1.34C	198.6	79.6	182.3	75.6
1.0	2.69C	179.0	71.7	159.7	66.2
2.0	5.38C	154.5	61.9	130.1	54.0
0.1 (recovery)	0.27C	290.9	116.5	266.8	110.6

* C-rate calculated relative to the theoretical capacity of graphite (1C = 372 mA g^−1^).

**Table 5:** Equivalent-circuit parameters and Warburg coefficients of the LDPE-C-350/900 and HDPE-C-350/900 electrodes after the 3rd, 50th and 100th cycles.

Electrode	Cycle	R_s_ (Ω)	R_SEI_ (Ω)	R_ct_ (Ω)	R_SEI_ + R_ct_ (Ω)	σ (Ω s^−1/2^)	χ^2^
LDPE-C-350/900	3	1.13 ± 0.08	34.97 ± 0.70	13.13 ± 1.51	48.10	24.7	0.106
	50	3.48 ± 0.15	17.15 ± 0.55	11.40 ± 0.51	28.55	18.3	0.188
	100	1.65 ± 0.45	17.63 ± 2.29	7.96 ± 0.46	25.59	14.5	0.137
HDPE-C-350/900	3	5.81 ± 0.07	47.99 ± 0.41	3.53 ± 0.44	51.52	23.9	0.036
	50	2.94 ± 0.11	17.27 ± 0.71	4.19 ± 0.72	21.46	16.6	0.203
	100	5.00 ± 0.30	12.35 ± 0.96	3.10 ± 0.27	15.45	15.9 ^a^	0.117

Equivalent circuit: R_s_ + (R_SEI_‖CPE_SEI_) + (R_ct_‖CPE_dl_) + CPE_W_. Errors are the standard errors of the fit (EC-Lab ZFit, Monte-Carlo, |Z|^2^ weighting); χ^2^ is χ^2^/|Z|^2^. σ is the slope of Z′ vs. ω^−1/2^ in the range 0.01–0.1 Hz (R^2^ ≥ 0.999). ^a^ The spectrum ends at 20 mHz, so σ is obtained from 8 points (R^2^ = 0.989).

**Table 6:** Electrochemical characteristics of the present carbons compared with carbons from other waste feedstocks in lithium half-cells. Values are as reported in the cited works; test conditions differ between studies.

Precursor	Treatment	Reversible Capacity (mA h g^−1^)	Rate Capability	ICE (%)	Ref.
Waste LDPE/HDPE	Solvothermal sulfonation	230/350	n.r.	n.r.	[13]
PP disposable masks	Sulfonation	438.1 (max. discharge)	56.5% retained at 5C	48.4	[48]
Waste plastic bags	Autogenic reaction	240 at 1C	n.r.	≈40	[49]
Waste PP packaging	H_2_SO_4_ cross-linking	373.1 at 0.1 A g^−1^ (100th cycle)	111.1 mA h g^−1^ at 2 A g^−1^	30.2–46.7	[10]
ABS 3D-printing waste	H_2_SO_4_ cross-linking	>300 at 20 mA g^−1^ (100th cycle)	tested 20–2000 mA g^−1^	≈67	[11]
Olive pomace (biomass)	H_3_PO_4_ activation	288 at 25 mA g^−1^ (70th cycle)	n.r.	n.r.	[9]
Hard carbons (typical)	—	—	—	50–80	[38]
Waste LDPE film	H_2_SO_4_ sulfonation	337.5 at 0.1 A g^−1^ (100th cycle)	154.5 mA h g^−1^ at 2 A g^−1^ (61.9%)	55.6	this work
Waste HDPE bottle	H_2_SO_4_ sulfonation	302.2 at 0.1 A g^−1^ (100th cycle)	130.1 mA h g^−1^ at 2 A g^−1^ (54.0%)	63.1	this work

n.r. = not reported. ICE = initial coulombic efficiency.

## Data Availability

The original contributions presented in this study are included in the article. Further inquiries can be directed to the corresponding author.

## Abbreviations

The following abbreviations are used in this manuscript:

PE	Polyethylene
LDPE	Low-density polyethylene
HDPE	High-density polyethylene
LIB(s)	Lithium-ion battery(-ies)
SEM	Scanning electron microscopy
HRTEM	High-resolution transmission electron microscopy
XRD	X-ray diffraction
XPS	X-ray photoelectron spectroscopy
TGA	Thermogravimetric analysis
BET	Brunauer–Emmett–Teller
2D-NLDFT	Two-dimensional non-local density functional theory
EIS	Electrochemical impedance spectroscopy
NMP	N-methyl-2-pyrrolidone
PVDF	Poly(vinylidene fluoride)
AB	Acetylene black
EC	Ethylene carbonate
DEC	Diethyl carbonate
DMC	Dimethyl carbonate
CV	Cyclic voltammetry
ICE	Initial coulombic efficiency
CE	Coulombic efficiency
SEI	Solid electrolyte interphase
dQ/dV	Differential capacity (derivative of charge with respect to voltage)
FWHM	Full width at half maximum
d_002_	Interlayer spacing (002 reflection)
L_c_	Crystallite dimension along the c-axis
L_a_	Crystallite dimension along the a-axis
N	Average number of stacked layers
A_D_/A_G_	Raman D-band to G-band integrated intensity ratio
R_s_, R_SEI_, R_ct_	Series, SEI and charge-transfer resistances
σ	Warburg coefficient
CPE	Constant phase element
SAED	Selected-area electron diffraction
